# The Radicalization of Brexit Activists

**DOI:** 10.3389/fpsyg.2021.798232

**Published:** 2022-02-09

**Authors:** Clare B. Mason, David A. Winter, Stefanie Schmeer, Bibi T. J. S. L. Berrington

**Affiliations:** Department of Psychology, Sport and Geography, University of Hertfordshire, Hatfield, United Kingdom

**Keywords:** Brexit, activist, radicalization, constructivist, identity fusion, extreme pro-group behavior, repertory grid

## Abstract

Brexit activists demonstrating outside the British Houses of Parliament were studied *in situ* to examine their potential for pro-group extreme behavior. This involved activists of two polarized, opposing views; those of Leave and Remain. The research engaged concepts linking the different theoretical perspectives of identity fusion and personal construct psychology. The study measured participants' degree of fusion to their group using a verbal measure. Willingness to undertake extreme acts was assessed in several ways: a measure of willingness to fight for the group, adaptations of the trolley dilemma and questions regarding political violence. Individual construing was examined using repertory grid technique and a semi-structured interview. Results were similar for both Leave and Remain participants. The majority of activists identified as “fused” to their group and, if so, were more likely to undertake hypothetical extreme behavior compared to those who did not identify as “fused.” Repertory grid technique indicated that becoming an activist provided individuals with a clearer and more positive view of themselves. Opposition activists were construed more negatively and extremely than fellow activists, and this construal was associated with an increased willingness to undertake extreme pro-group behavior. This was consistent with the personal construct model of radicalization and was heightened in those who were “fused.” Interview data provided support for the constructivist model and revealed characteristics and concerns of the two groups. Overall, the findings indicate that campaigning organizations contain fused individuals, who are more likely to undertake hypothetical pro-group violence including self-sacrifice. This has broader implications which may be particularly pertinent, given the violent impact of extremist activists around the globe.

## Introduction

From the 2016 referendum to its official departure from the European Union (EU) in 2020, the United Kingdom was characterized by passionate and divisive arguments. Politicians were labeled as “heroes” or “traitors,” friends became “ex-friends,” even close family ties were stretched to breaking point and it was unsurprising that “Leave” and “Remain” demonstrations often escalated into aggressive and violent clashes as a result of such impassioned dispute and the emergence of partisan groups. But what leads an individual to undertake violent actions on behalf of their group? The question reaches beyond Brexit, extending to other activist groups motivated by strongly held beliefs. This includes the actions of animal rights activists, far-right extremists and the faith-based extremism of individuals involved in devastating acts of terrorism.

In this study, the identity fusion and personal construing of individuals demonstrating for and against Brexit are explored. Their willingness to undertake acts of extreme behavior on behalf of the group is also examined.

On 24 June 2016, after a fiercely fought campaign, Britain voted to leave the European Union by a majority of just 4%. This resulted in a bitterly divided nation and political paralysis.

Prime Minister David Cameron resigned as a result of the referendum, after which the subsequent Prime Minister, Theresa May, launched a two-year process of departure from the EU by triggering Article 50^1^ on 29 March 2017. However, with insufficient parliamentary support, Mrs. May was forced to request an extension to the country's withdrawal proposals on three occasions before finally resigning on 24 July 2019. The following Prime Minister, Boris Johnson, called a General Election on 12 December 2019, winning an increased parliamentary majority and committing to leaving the EU swiftly. At 11 p.m. on 31 January 2020, almost 4 years after the referendum, the United Kingdom departed the European Union.

During the prolonged Brexit period, the hopes and anxieties of campaign groups were raised and dashed at each departure date and postponement. Members of Parliament were vilified for voting counter to their constituency majorities. Demonstrators outside the Houses of Parliament increased in number and displayed increasing animosity toward one another. Aggressive verbal abuse and violent scuffles between opposing activists, as witnessed by the first author, increased in frequency and vehemence. Whilst much of this was at a minor level, violent action on behalf of the group suggested commonality with the process of radicalization.

For many, radicalization is synonymous with terrorism. However, it is a process involving a progression of thought. Individuals can be found at various points along this pathway, including non-violent stages (e.g., Borum, 2003; Wiktorowicz, 2005; Moghaddam, 2009; Winter and Feixas, 2019).

During the Brexit campaign, violence was used by a small number of activists in an attempt to achieve their goal by defending an opinion (Busby, 2019) or by intimidating a minority (e.g., Burnett, 2017; Rzepnikowska, 2019). This is in line with the EU definition of radicalization as “a phenomenon of people who regard the use of violence as legitimate and/or use violence themselves in order to achieve their political objectives which undermine the democratic legal order and the fundamental rights on which it is based” (European Union Committee of the Regions, 2016, p. 4). Thus, whilst Leave and Remain are far from terrorist organizations, the process by which some campaigners became violent may follow the same pathway as an individual who progresses further, to acts of devastating terrorism.

Identity fusion theory and personal construct psychology provide a useful and novel theoretical framework to investigate this phenomenon.

Social identity theory (Tajfel, 1978; Tajfel and Turner, 1979) proposes that a person's sense of self involves the groups they belong to. It distinguishes between personal identities as those involving individual qualities, such as kindness, and social identities as those referring to groups, such as nationality. For most, there is a clear distinction between the two. However, Swann et al. (2009) propose that for some individuals, these identities fuse together. While social identity theory suggests that the salience of one type of identity decreases when the other is strong, the identity fusion approach proposes that although fused, both identities remain strong, responding in a synergistic manner to produce exceptional investment in the group. This can manifest in personally costly, pro-group behaviors, including self-sacrifice (e.g., Swann et al., 2010a) and fighting for the group (e.g., Gómez and Vázquez, 2015). It can also be predictive of altruistic acts such as rushing to the aid of bomb victims (Buhrmester et al., 2015). Identity fusion is readily seen in familial relations (Vázquez et al., 2019) but is also observed in many collective groups, even where the individual is unacquainted with the majority of their members. This includes political movements where group members recognize that others share their core characteristics, making them appear “family like” and, potentially, worth dying for (Swann et al., 2014a), such as the Brexit campaigns.

Personal Construct Psychology (PCP) was devised by the American psychologist George Kelly. In essence, it proposes that individuals are like scientists. They continually devise, test and revise personal theories to understand their world and anticipate future experiences (Kelly, 1955). Construct systems (the “theories”) are comprised of personal constructs that are bipolar in nature. For example, “good” has meaning when related to “bad.” An individual will place “elements,” such as people at different points along each of their constructs, depending on their experiences of the person concerned. This enables an anticipation and understanding of people and their behavior. Constructs are arranged in hierarchies with superordinate constructs subsuming those that are subordinate. For example, “good—bad” may subsume “intelligent—stupid.” If subsequent experiences challenge, or invalidate, the individual's predictions, they will generally revise these. For example, if an individual perceived as “good” later verbally assaults someone, the individual might be reconstrued as “bad.” However, after several such experiences, the “good—bad” construct itself might need to be revised. In this way, individuals are able to continue to understand and predict the world around them. However, sometimes invalidation can be immensely problematic, particularly if it affects an individual's core constructs, which are those which embody fundamental values, a sense of self and identity. This is thought to occur in those who become radicalized.

The constructivist model of radicalization (Winter and Feixas, 2019) provides the guiding theoretical framework to this study. It describes several stages to radicalization, as outlined below. With its basis in how the individual views, anticipates and responds to the world, this model is able to accommodate the concepts of other pathways to and models of radicalization (Winter and Feixas, 2019, p. 3–5).

1. *The radicalized individual has a history of invalidation of his/her construing, particularly in regard to core aspects of self-construing***. **This leads the individual to a state of uncertainty, a factor recognized by other authors as being linked to radicalization (e.g., Hogg et al., 2013).
2. *Invalidation can sometimes involve one or more episodes that lead to massive invalidation, and act as “transformative triggers.”* This occurs when several superordinate structures, including core constructs, become invalidated in a short period of time. The resultant extreme uncertainty is experienced as intense anxiety, threat and associated emotional responses.
3. *The individual with a very undifferentiated (and thus inflexible) construct system may be particularly vulnerable to such invalidation and consequent structural collapse***. **Individuals with undifferentiated (inflexible) construct systems have a limited view of events. Their construct system cannot easily provide an alternative understanding and they may be particularly vulnerable to construct invalidation.
4. *His/her radical beliefs, usually drawing upon available social constructions, allow the development of a “turning point” in his or her sense of identity with a more structured and certain view of the world*. Following attempts to reconstrue in order to understand their experience, an individual may turn to an ideological framework to restore certainty, reduce anxiety and have a new core role as a member of the group.
5. *The development of an extreme negative construction of another group, which may be perceived as responsible for the individual's invalidations, allows further definition of the self by contrast with this group*. The negative construing of the out-group facilitates a positive view of the self.
6. *The individual's radical constructions are validated by contact with others who share similar views, often coupled with constriction of their previous social world to avoid further invalidation*. Radicalized individuals will often reduce their social contacts to those who are their primary source of validation.
7. *The likelihood of acting upon radical beliefs, including violent actions, is greater in those individuals in whom beliefs in such actions provide the greatest increment in the structure of his/her view of the self* . Taking extreme actions may enhance the structure and certainty provided by their new role.
8. *Reconstruing of violence as acceptable may be necessary if the person is to engage in such acts without guilt (and indeed to experience guilt for not engaging in them)*. It is possible for violence to be reconstrued as a legitimate form of action by the group. It may even be seen as essential in a “supreme” goal.
9. *His/her radical view of the world may be shored up by “hostility,” in Kelly's (**1955**) sense of extorting evidence for the individual's constructions*. Kelly (1955) describes hostility as when an individual is unable to revise their construct system to understand new events and instead forces the evidence to fit. In this way, radicalized and extremist views are maintained despite invalidations from others, that is, the majority of society.
10. *Similar processes may operate in members of the “other” group, creating a vicious cycle of extreme construing based on mutual validation of extreme negative views of the other*.

Using a novel combination of PCP and identity fusion measures, the study aimed to examine the construing of Leave and Remain activists demonstrating outside the British Houses of Parliament. These dichotomous, polarized groups were expected to demonstrate typical and possibly extreme group dynamics, making them a pertinent study population. Significantly, the study focused on “real-world” activists, rather than students or laboratory-based participants. It investigated whether Brexit activists were fused to their group and if this influenced their willingness to undertake hypothetical extreme and violent pro-group behaviors. It also examined whether their construing was consistent with the constructivist model of radicalization.

The research extends the work of Swann et al. (2009) and Winter and Feixas (2019), by exploring the psychological processes of individuals demonstrating potential extremist activity, thus possibly allowing the development of an approach to help predict such behaviors.

## Materials and Methods

### Participants

Sixty-five Brexit activists participated in the study (38 males, 27 females; age ranging from 23 to 80 years, *M* = 56.30, *SD* = 12.2). All had traveled to central London to demonstrate. Sixty-two (95%) were of British nationality (including dual citizenship), one was a non-British EU citizen, and two were non-British, non-EU citizens. Regarding political campaigning, 37 participants (57%) campaigned to remain in the European Union and 28 participants (43%) campaigned to leave. The greater number of Remain participants (57%) was an outcome of opportunity sampling, reflecting the difference in Leave and Remain numbers present at Brexit demonstrations (Mills, 2019).

### Materials

#### Identity Fusion

The study measured participants' degree of fusion to their campaign group using the verbal measure of Gómez et al. (2011). This involved responding on a scale ranging from 1 to 7, strongly agree to strongly disagree, to a series of seven questions regarding their relationship with fellow activists. Examples of the questions include “I am one with other [Leavers/Remainers];” “I feel immersed in the [Leave/Remain] group;” “I make the [Leave/Remain] group strong.”

#### Willingness to Undertake Extreme Acts on Behalf of the Group

In order to explore a hypothetical willingness to undertake extreme acts on behalf of the group, participants were presented with a series of adapted measures:

**Questions Regarding Political Violence (adapted from Ginges and Atran**, **2011****)**. Participants were asked to respond with “I would do; I might do or I would never do” to the following questions:

*Would you engage in political violence (including damage to property and persons) if [Leave/Remain] was to be forcibly dismantled before Brexit took place?*

*Would you engage in political violence (including damage to property and persons) if it would gain the political change [Leave/Remain] are campaigning for?*

**Trolley Dilemma**. Two adaptations of the trolley dilemma (Foot, 1967; Swann et al., 2010a) were presented with graphical representation alongside the text.

1. Self-sacrifice to save five in-group members: Participants could choose to (a) do nothing and let a runaway trolley kill 5 fellow activists, or (b) sacrifice their life by jumping onto the track of the trolley to save the five fellow activists.
2. Self-sacrifice to save five out-group members or one in-group member: Participants could choose to (a) observe the situation, (b) sacrifice their life by jumping onto the track to divert the trolley to save five opposition activists (resulting in the death of one fellow activist), or (c) sacrifice their life by jumping onto the track to divert the trolley to save one fellow activist (resulting in the death of five opposition activists).

**Measure of Willingness to Fight for the Group** (Swann et al., 2010b). This involved responding on a scale ranging from 1 to 5, strongly agree to strongly disagree, to a set of seven questions regarding the participant's willingness to fight for the group. Examples of the questions include: “I would fight someone who was physically threatening another [Leaver/Remainer];” “Hurting other people is acceptable if it means protecting [Leavers/Remainers];” “I would sacrifice my life if it saved another [Leaver/Remainer]'s life.”

#### Individual Construing

**Repertory Grid**. To examine their individual construct systems, participants were presented with a Repertory Grid (Kelly, 1955) adapted by Winter (2011) for the study of radicalization. Participants rated a supplied set of elements (people) against a series of bi-polar constructs. The elements were: self before and after becoming a [Leave/Remain] activist; ideal self; three fellow [Leave/Remain] activists (known to them personally or in the public arena); three opposition [Remain/Leave] activists (known to them personally or in the public arena); and a neutral individual (someone who is not interested in Brexit, known to them personally or in the public arena). Twelve bi-polar constructs were elicited by asking participants to make a distinction between successive triads of elements. That is, participants were asked an important way in which two of the elements (people) in each triad were similar and thus differed from the third. On a scale ranging from 1 to 7, participants then rated each element (person) on each construct, for example, how politically engaged—politically not engaged they considered a fellow activist to be.

**Figure 1:**
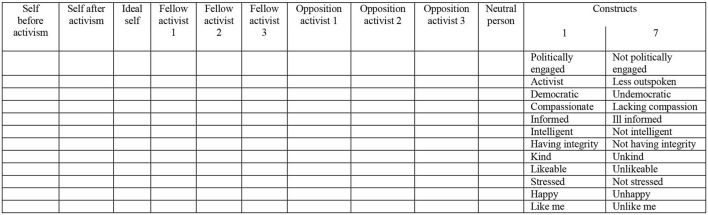
Repertory grid (adapted from Winter, 2011).

Standardized grids containing pre-defined elements and constructs enable direct comparison between individuals and groups. As responses from the first eight participants demonstrated a high commonality in constructs, a standardized grid was produced using the most frequently elicited constructs, together with the construct “like me—unlike me” (Figure 1). Results reported refer to the standardized grids. As six of the initial eight participants also completed the standardized grid, the total number of participants was 63.

Analysis of repertory grid data was undertaken using the software programmes IDIOGRID (Grice, 2002) and GRIDSTAT (Bell, 2009). The measures derived were as follows (see also Grice, 2002):

i. *Correlation of Average Grids*. Average grids were produced for each group by IDIOGRID, which then provided a measure of general degree of correlation between the two average grids.
ii. *Tightness of Construing*. Principal component analysis of the repertory grid conducted by IDIOGRID provided an indication of tightness, or lack of differentiation, in construing. A higher value for the variance accounted for by the first principal component suggests a less flexible, more rigid and stereotyped way of thinking.
iii. *Distance from Ideal Self*. High scores on a measure of element distance provided by IDIOGRID indicate a construed dissimilarity of an element (person) from the ideal self, in other words a more negative construing of that particular element (person).
iv. *Salience of Fellow and Opposition Activists*. Measured by percentage sum of squares of the ratings provided for fellow and opposition activists in the repertory grid, again derived from IDIOGRID, higher scores indicate that the element (person) concerned holds more meaning for the individual and is likely to be construed more extremely.
v. *Conflict in Construing*. This refers to a logical inconsistency in construing, as defined by Bell (2004). The percentage of conflict in construing associated with different elements was established using the software programme GRIDSTAT (Bell, 2009).
vi. *Discriminatory Capability of Constructs*. The discrimination between elements (people) that each construct is responsible for is measured by IDIOGRID by the percentage of the sum of squares accounted for by the construct (Higher scores indicate higher discriminatory capability and greater usefulness of the construct).

These measures provide an indication of:

- similarity in the construing of different groups (measure i);
- tightness of construing (measure ii);
- the relative positive/negative construing of the self before and after becoming an activist (measure iii);
- the relative positive/negative construing of fellow and opposition activists (measure iii);
- the relative salience (meaningfulness) of fellow and opposition activists (measure iv);
- the relative amount of conflict associated with becoming an activist (measure v);
- the relative amount of conflict associated with fellow and opposition activists (measure v);
- the relative usefulness of constructs (measure vi).

**Interview**. All participants were invited to undertake a semi-structured interview which was centered around the following questions:

*Why did you become a member of the [Leave/Remain] campaign group?*

*What life experiences led you to join?*

*What changes have you experienced since becoming a member of [Leave/Remain] group?*

*Have you witnessed or been involved in any violent incidents whilst campaigning?*

*How close do you feel to your fellow [Leave/Remain] group members?*

The interview transcripts were analyzed using a hybrid deductive and inductive approach as described by Fereday and Muir-Cochrane (2006), adapted to incorporate Braun and Clarke's (2006) Thematic Content Analysis methodology. This approach enabled a search for evidence of the constructivist model whilst also allowing for themes to emerge directly from the data. For the deductive approach, a code book was developed a priori. Codes and sub-codes were established for each stage of the constructivist model. These were labeled, defined and a description provided of how they may present in the text. To ensure the applicability and rigor of the code book, an interview transcript was selected as a test piece and independently coded by two of the authors. The results were compared and no modifications to the predetermined code book were required. In the inductive thematic content analysis approach, codes were developed as they emerged from the data, and again reviewed by two researchers for rigor. The emergent themes would provide an indication of the characterization and concerns of the two groups.

### Procedure

The study took place during the later stages of the Brexit process, from February 2019 until the United Kingdom left the European Union on 31 January 2020. Participants were approached by a female experimenter whilst actively campaigning outside the Houses of Parliament in Westminster, London. Participants completed the study *in situ*. Privacy and confidentiality were provided by appropriately distancing from others.

## Results

### General Analytical Approach

Analysis was undertaken to identify participants who identified as “fused” according to the measure of Gómez et al. (2011). Previous studies (e.g., Swann et al., 2009) indicated that, compared to non-fused participants, those who were fused would be more willing to undertake hypothetical acts of extreme pro-group behavior and self-sacrifice on behalf of the group. Noting that effects are more apparent in strongly fused subjects (Swann et al., 2014b), those in the mildly fused category were excluded from comparative analyses.

Parametric and non-parametric tests used to examine group differences were *t*-tests (independent and paired samples) and Mann-Whitney *U* and Wilcoxon tests respectively. Kendall's tau was used to examine relationships between measures where data were not normally distributed. Where cell frequencies fell below the required threshold, Fisher's exact test was used as an alternative to chi-squared. Non-parametric tests were used where conditions for parametric tests were not met.

Fusion “categories” were determined through mean item scores of the identity fusion verbal measure (Gómez et al., 2011):

Not fused < 5.0
Fused mild ≥ 5.0 and < 6.0
Fused moderate ≥ 6.0 and < 7.0
Fused strong = 7.0.

### Study Population in Terms of Identity Fusion and Campaign Group

The proportion of study participants described as fused (mild to strong) was substantial, 71% of the study population, with similar totals occurring in both Leave and Remain groups. However, a difference appeared on first observation of the fusion subcategories (Table 1). The Leave population appeared to have a higher proportion who were moderately fused, and were the only participants appearing in the strongly fused category. In contrast, a larger proportion of Remain participants appeared in the mildly fused category. However, these differences were not found to be statistically significant, χ^2^ = 3.22 (*p* > 0.05) and no statistically significant difference was observed between the identity fusion scores of Leave (*Mdn* = 41.00) and Remain (*Mdn* = 40.00), U(NLeave = 27, NRemain = 38) = 423.50, *z* = −1.28, *p* = 0.21 (two-tailed).

**Table 1:** Identity fusion category according to Brexit referendum vote.

**Group and fusion category**	***n***	**As proportion of total population (*N* = 65)**	**As proportion of Leave (*n* = 28) or Remain (*n* = 37) subsample**
**Fused (all)**			
Total	46	70.8%	-
Leave	19	29.2%	67.9%
Remain	27	41.5%	73.0%
**Fused (moderate** **+** **strong)**			
Total	22	33.8%	-
Leave	12	23.1%	53.6%
Remain	10	15.4%	27.0%
**Fused (moderate)**			
Total	19	29.2%	-
Leave	9	13.9%	42.9%
Remain	10	15.4%	27.0%
**Fused (strong)**			
Total	3	4.6%	-
Leave	3	4.6%	10.7%
Remain	0	0.0%	0.0%
**Fused (mild)**			
Total	24	36.9%	-
Leave	7	10.8%	25.0%
Remain	17	26.2%	45.9%

### Extreme Pro-group Actions

#### Willingness to Undertake Extreme Behavior on Behalf of the Group

**Figure 2:**
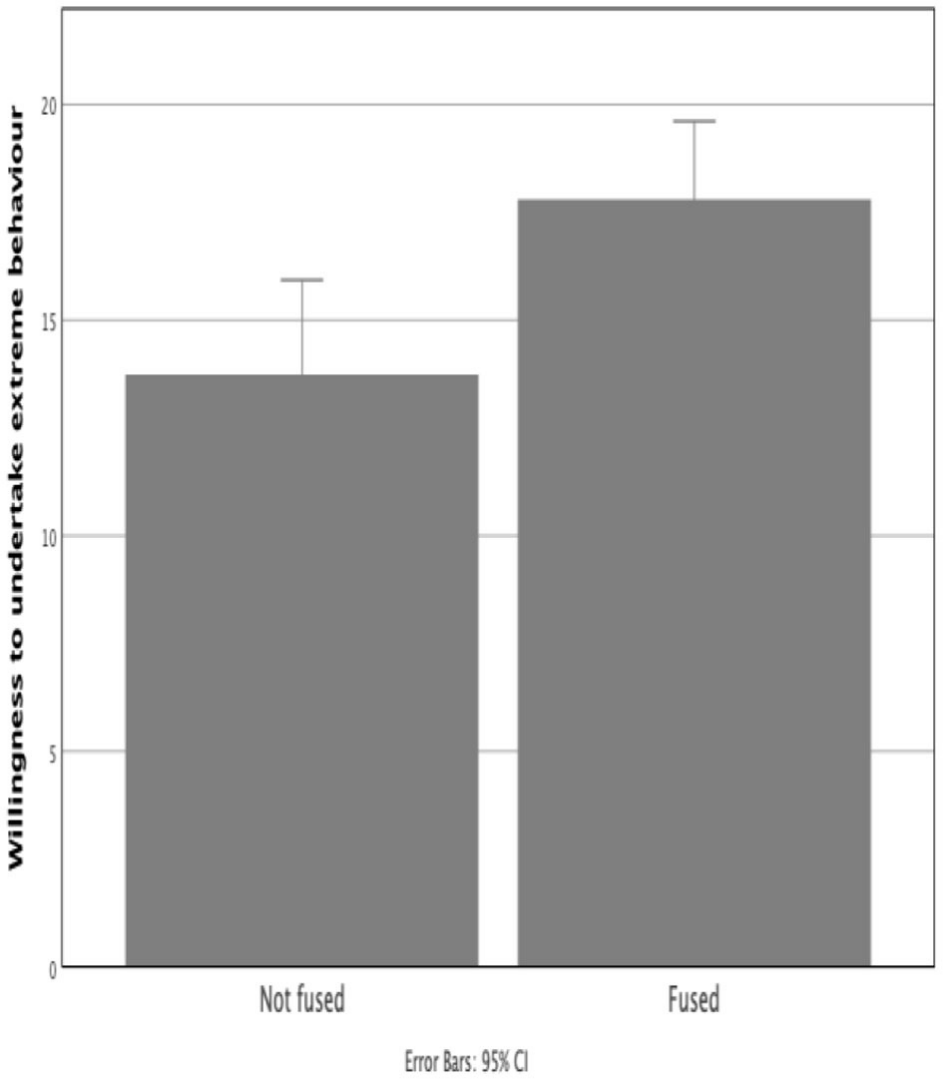
Measure of willingness to undertake extreme pro-group behavior (Swann et al., 2010b).

Fused participants (fusion measurement score, Gómez et al., 2011) (*M* = 19.09, *SD* = 7.06) were significantly more willing than non-fused participants (*M* = 13.74, *SD* = 4.56) to undertake hypothetical extreme acts on behalf of the group (according to the measure of Swann et al., 2010b), *t*(63) = 2.84, *p* < 0.001 (Figure 2). This was observed to a greater extent in the Leave subgroups, Leave Fused (*M* = 21.92, *SD* = 7.03) and Non-fused (*M* = 12.22, *SD* = 4.21), *t*(19) = 3.66, *p* < 0.001. Results for Remain were not significant. This may be due to the relatively smaller proportion of Remain activists in the moderate-strong fusion category.

#### Predicting Extreme Pro-group Behavior

Linear regression analysis indicated a significant model for the positive relationship between fusion measure scores (Gómez et al., 2011) and willingness to undertake extreme behavior (Swann et al., 2010b), *F*(1, 63) = 15.93, *p* < 0.001. The fusion score accounted for 19% of the variability in willingness to undertake extreme behavior. The regression equation was: Extreme pro-group behavior score = 1.53 + (2.76 × fusion score). In summary, fusion score (Beta 0.45, *p* = < 0.001) significantly predicted a willingness to undertake extreme behavior.

#### Sacrificing One's Life to Save Fellow Activists

The analysis of responses to the first adapted trolley dilemma suggested that the likelihood of (hypothetically) sacrificing one's life to save five fellow activists (rather than doing nothing) was somewhat greater for fused participants than non-fused participants [*p* < 0.05 (one-tailed), Fisher's exact test].

The effect was enhanced in the second trolley dilemma where fused participants declared that they were even more likely to sacrifice their life to save just one fellow activist when there were also options to either save five opposition activists or do nothing [*p* < 0.001, (one-tailed), Fisher's exact test]. In this case, there is a heightened willingness to sacrifice one's life for a fellow group member, when faced with the alternative of saving opposition activists. This suggests that the pre-eminence of one's group is heightened in the presence of the opposing group.

#### Participation in Political Violence (Including Damage to Persons and Property)

Fused participants were somewhat more likely than non-fused participants to indicate that they might or would engage in political violence if the group were forcibly dismantled [*p* < 0.05 (one-tailed), Fisher's exact test].

In comparison, fused participants were not more likely than non-fused participants to indicate that they might or would engage in political violence to achieve the group's aims. This suggests that for fused participants there is greater importance attached to group membership than its goals.

### The Construing of Leave and Remain Activists (From Analyses of Repertory Grid Data)

#### Correlation of Average Grids

**Figure 3:**
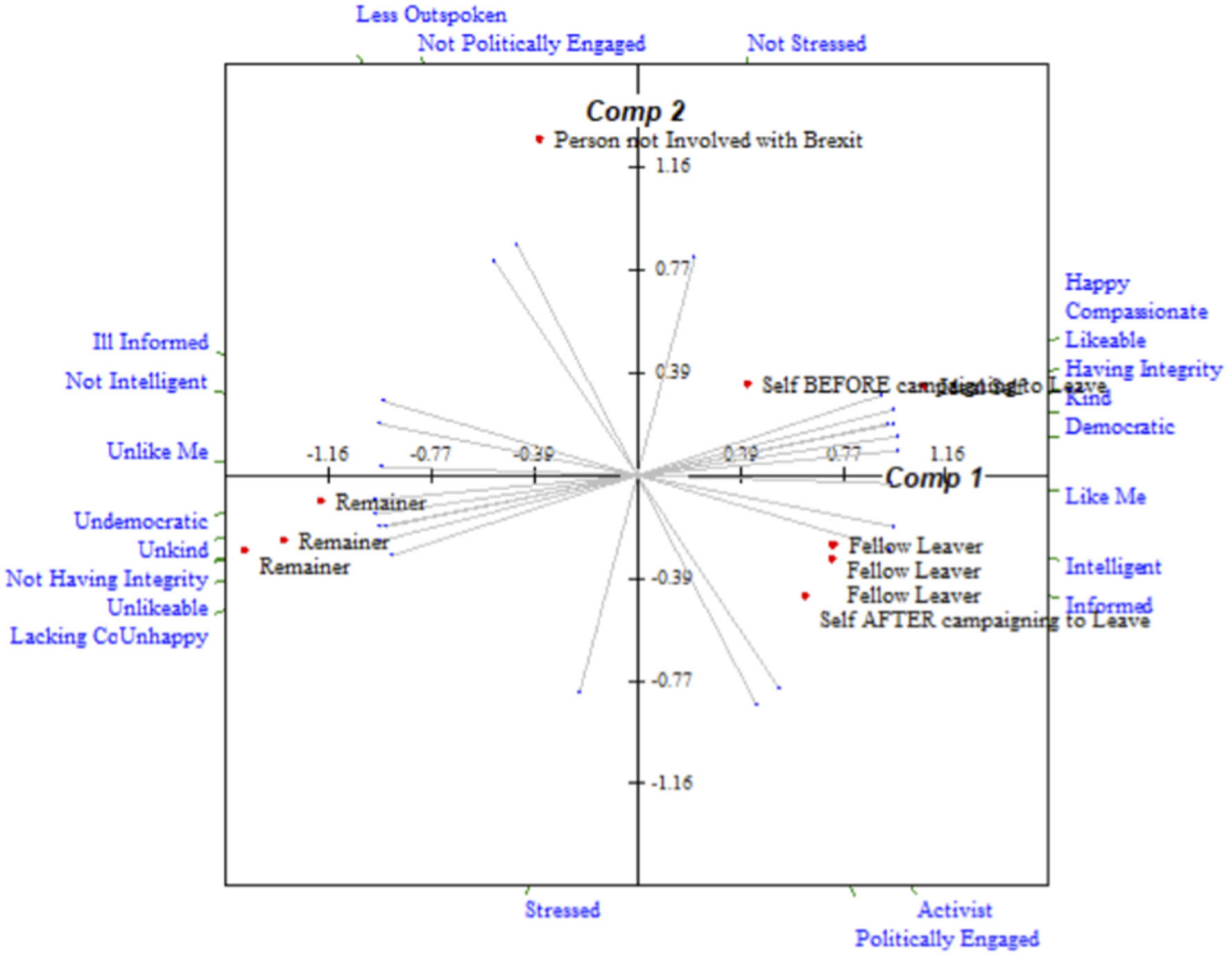
Plot of elements in construct space for Leave average grid.

**Figure 4:**
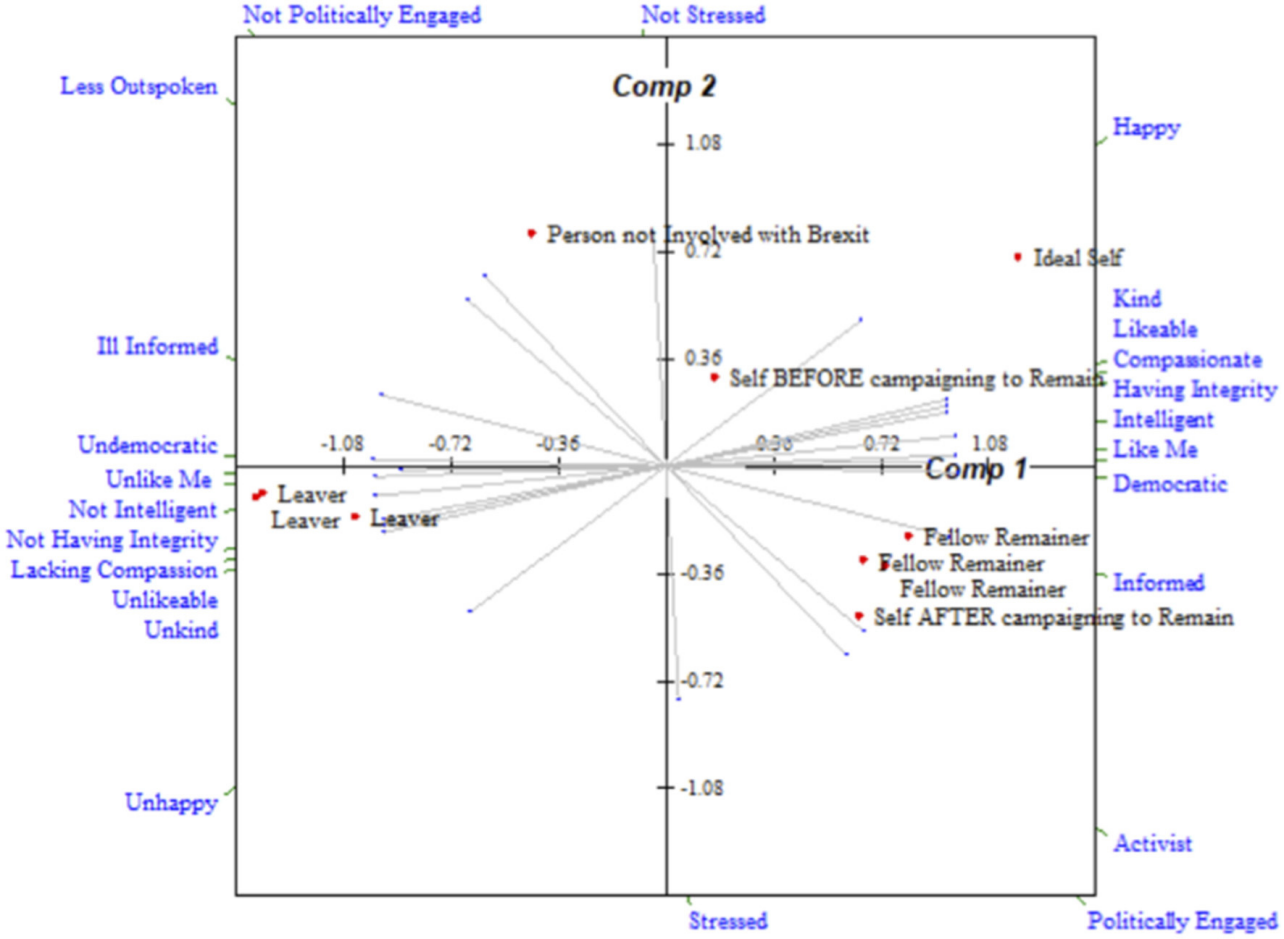
Plot of elements in construct space for Remain average grid.

The correlation between Leave and Remain average grids (calculated using IDIOGRID, Grice, 2002) highlights the similarity of the groups. Leave and Remain average grids were very highly correlated (general degree of correlation = 0.94), particularly the fused groups (general degree of correlation = 0.95). A lower correlation was observed between the non-fused groups (general degree of correlation = 0.82), perhaps reflecting the reduced group effect and reduced homogeneity of those who are not fused to a group. Plots of elements (people) in construct space derived from principal components analysis of the Leave and Remain average grids are shown in Figures 3, 4. These demonstrate the mirrored similarity of the two groups. Both groups see themselves as moving closer to their ideal self on becoming an activist. There is also a distinct and extreme difference in the positive and negative construing of fellow and opposition activists, respectively, for both Leave and Remain.

#### Tightness of Construing

The percentage of variance accounted for by the first principal component was similar for both groups, and there was no statistically significant difference between the two, Leave *M* = 63.33%, *SD* = 10.64, Remain *M* = 65.35%, *SD* = 8.26, *t*(60) = 0.82, *p* = 0.42 (two-tailed). This suggests that equally tight systems of construing were present in both of the groups involved in demonstrating in the Brexit debate.

#### Construing of the Self Before and After Activism

**Figure 5:**
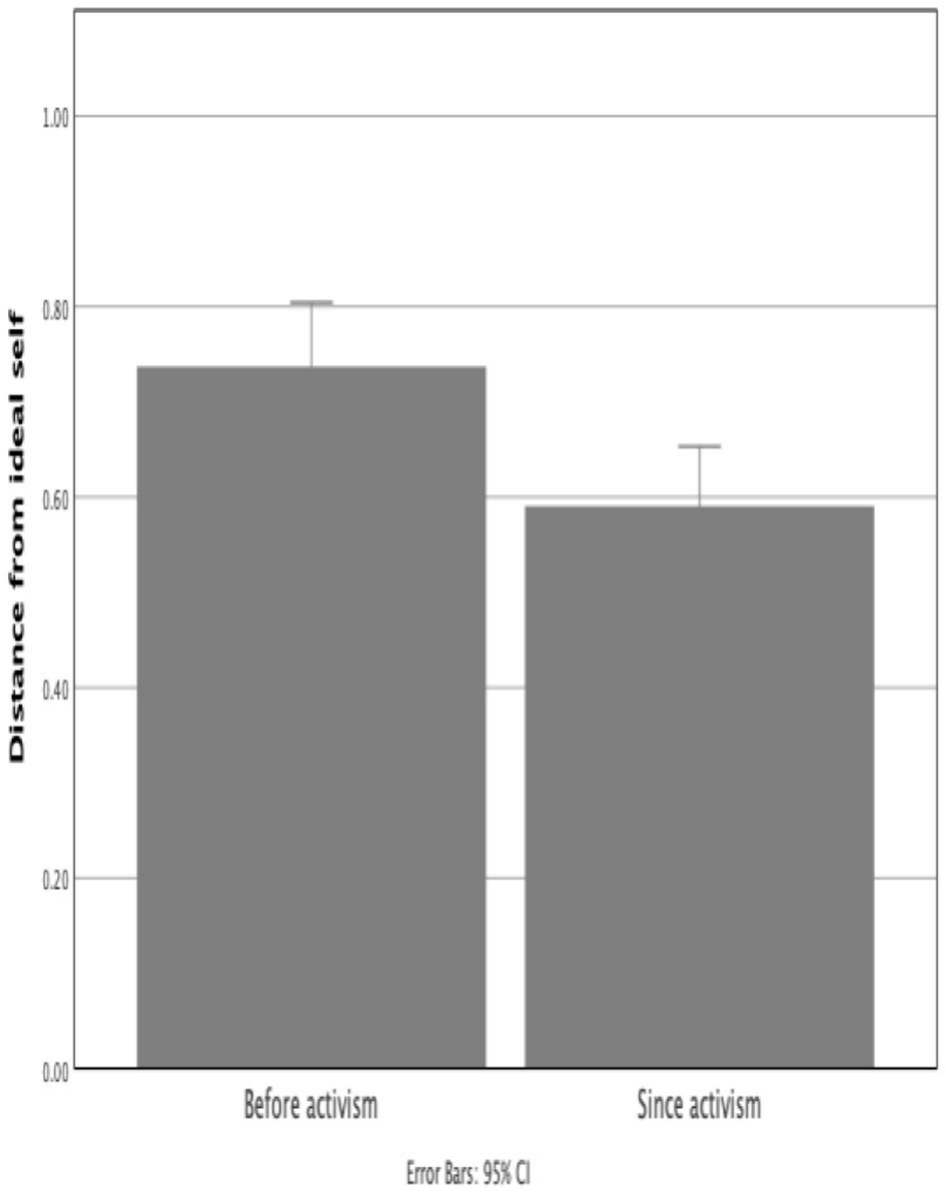
Distance from Ideal Self of participants before and after they become activists (smaller distances indicate a more favorable construing).

Element distance measures derived from the repertory grid allow measurement of how closely to their ideal people construe themselves. In this study, participants' responses suggested that becoming an activist brought them closer to their ideal self (Figure 5). That is to say, they construed themselves more positively after becoming an activist (*M* = 0.59, *SD* = 0.25) than before (*M* = 0.74, *SD* = 0.27), *t*(60) = 3.84, *p* < 0.001 (one-tailed).

#### Construing of Fellow and Opposition Activists

**Figure 6:**
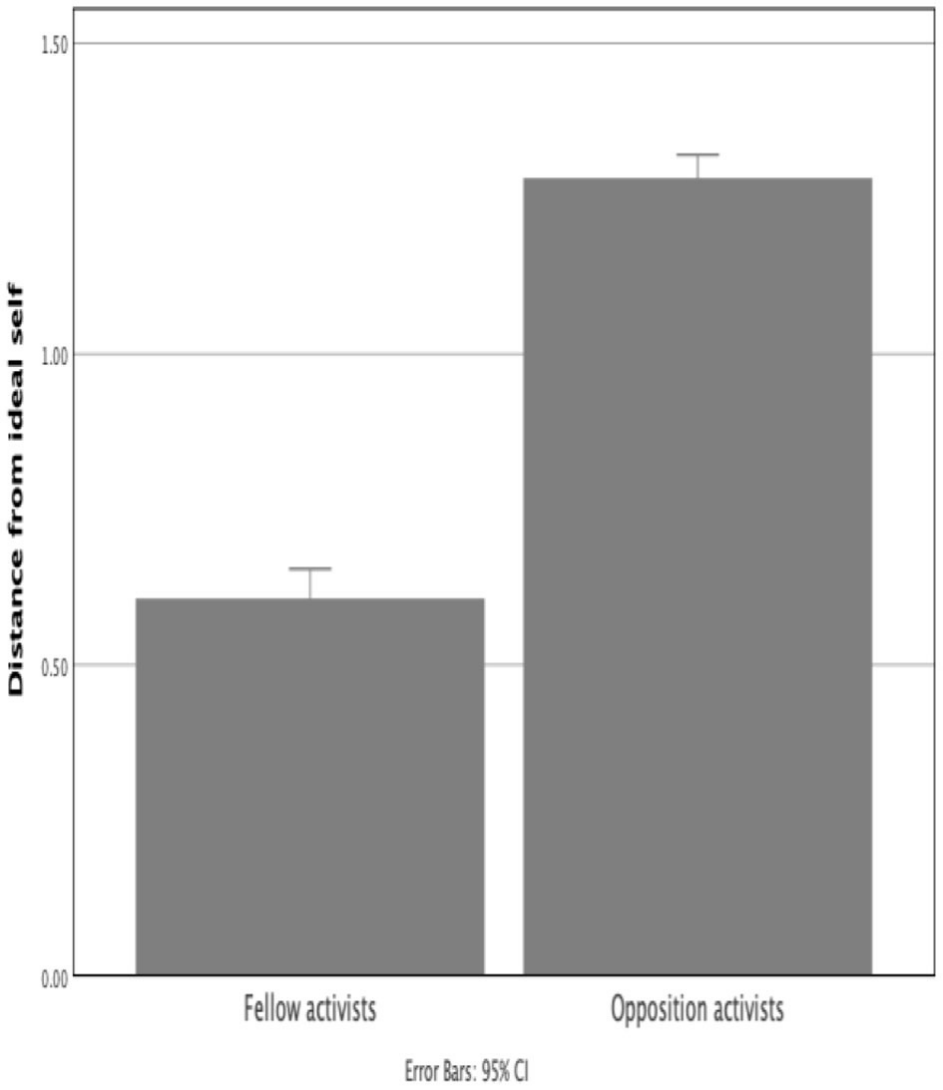
Distance from Ideal Self of fellow and opposition activists (larger distances indicate a more negative construing).

Unsurprisingly, opposition activists (*M* = 1.28, *SD* = 0.15) were construed in a more negative manner, as reflected in average distance from the ideal self, than fellow activists (*M* = 0.61, *SD* = 0.19), *t*(60) = 20.10, *p* < 0.001(one-tailed) (Figure 6 and Table 2). The magnitude of difference is notable, at ~2-fold throughout all groups.

**Table 2:** Distance from the Ideal Self of fellow and opposition activists by group (larger distances indicate a more negative construing.

**Group**	**Distance from Ideal Self**		**Significance of difference in distance from Ideal Self**	
	**Fellow activist**	**Opposition activist**		
	**Mean (SD)**	**Mean (SD)**	***t*** **-value**	***p*** **-value (1 tailed)**
**Total**				
Total	0.61 (0.19)	1.28 (0.15)	20.10	<0.001
Not fused	0.59 (0.20)	1.27 (0.15)	10.41	<0.001
Fused	0.63 (0.20)	1.32 (0.13)	11.77	<0.001
**Leave**				
Not fused	0.65 (0.20)	1.23 (0.13)	6.14	<0.001
Fused	0.63 (0.18)	1.31 (0.14)	9.05	<0.001
**Remain**				
Not fused	0.53 (0.20)	1.30 (0.17)	9.39	<0.001
Fused	0.64 (0.24)	1.35 (0.13)	7.19	<0.001

#### Salience of Fellow and Opposition Activists

**Figure 7:**
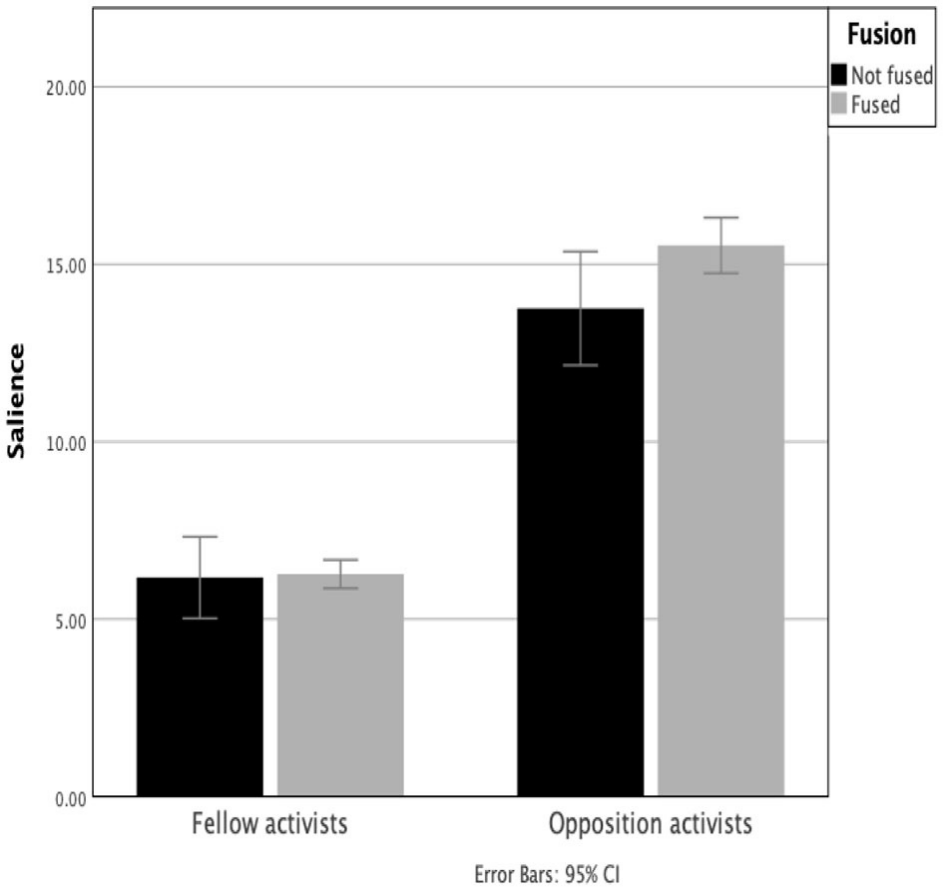
Salience of fellow and opposition activists by fusion category.

Opposition demonstrators (*M* = 15.02, *SD* = 2.87) were shown to be considerably more meaningful to, or extremely construed by, participants than their fellow activists, as indicated by the percentage sum of squares (*M* = 6.24, *SD* = 1.65), *t*(60) = 18.59, *p* < 0.001 (one-tailed). The difference was amplified in fused individuals (and shown to be statistically significant in both Leave and Remain groups, all *p's* < 0.001) (Figure 7 and Table 3). The greater values observed in fused participants appear to reflect an enhanced polarization of construing. The significance of the opposing political campaigner to the individual may be a contributory driver, or possibly a response, to becoming an activist. Politically neutral individuals were found to be less meaningful than opposition campaigners, *z*(60) = 2.32, *p* < 0.001 (one-tailed).

**Table 3:** Salience of fellow and opposition activists by group and fusion category.

**Group**	**Salience of fellow activists**	**Salience of opposition activists**		
	**Mean (SD)**	**Mean (SD)**	***t*** **-value**	***p*** **-value (1 tailed)**
**Total**				
Total	6.24 (1.65)	15.02 (2.87)	18.59	<0.001
Not fused	6.17 (2.31)	13.76 (3.22)	6.90	<0.001
Fused	6.41 (1.40)	15.29 (2.51)	12.91	<0.001
**Leave**				
Not fused	6.37 (2.58)	13.74 (2.68)	5.38	<0.001
Fused	6.81 (1.43)	14.79 (2.49)	8.68	<0.001
**Remain**				
Not fused	5.97 (2.15)	13.77 (1.28)	4.32	<0.001
Fused	5.88 (1.26)	15.95 (2.51)	10.67	<0.001

#### Conflict Associated With the Self Before and After Becoming an Activist

There was a decrease in conflict in construing associated with becoming an activist (*M* = 9.12, *SD* = 2.87), compared to the self before doing so (*M* = 9.80, *SD* = 3.36), *t*(60) = 3.84, *p* < 0.001 (one-tailed). In other words, becoming an activist provided a less conflicted and clearer sense of self.

#### Conflict Associated With Fellow and Opposition Activists

**Figure 8:**
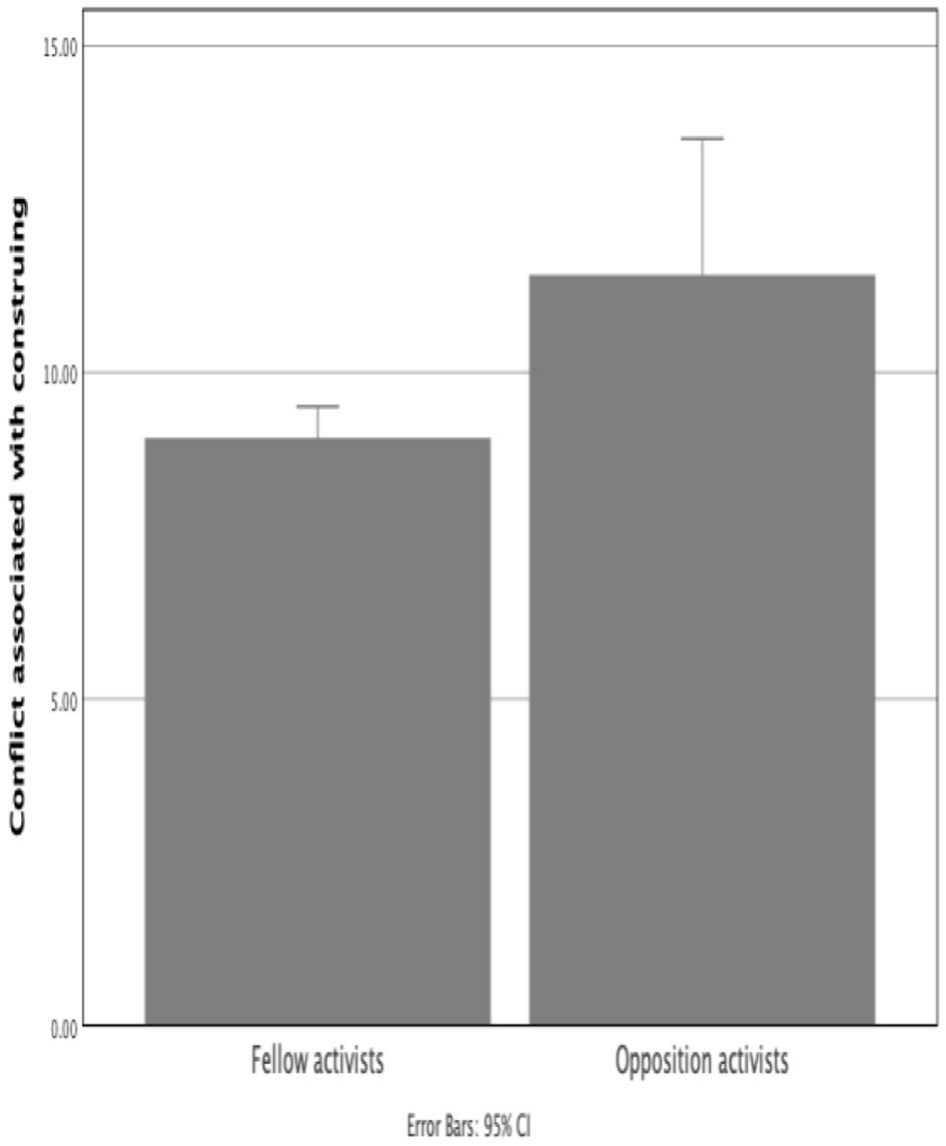
Amount of conflict associated with the construing of fellow and opposition activists. A lesser amount indicates a clearer and less conflicted way of construing.

A significantly smaller amount of conflict was associated with participants' construing of fellow demonstrators (*M* = 8.64, *SD* = 1.63) compared to opposition activists (*M* = 11.97, *SD* = 8.51), *z*(60) = 4.56, *p* < 0.001 (one-tailed) (Figure 8). This indicates that participants have a clearer and less conflicted way of construing those with similar than those with opposing beliefs. The large magnitude of difference may be unsurprising given the substantial differences observed in other measures (such as the valence of construing and salience of fellow and opposition activists).

#### Constructs

Using IDIOGRID (Grice, 2002), it is possible to determine which constructs are responsible for most discrimination between the elements (people) in a repertory grid, in other words, arguably these are the constructs that are most important to the individual (as explored below). Table 4 displays the constructs responsible for more than 9% of the total sum of squares (i.e., an above average amount in a grid with 12 constructs).

**Table 4:** Constructs responsible for most discrimination between elements (>9.00%).

**Leave**			**Remain**		
**Total**	**Not fused**	**Fused**	**Total**	**Not fused**	**Fused**
Democratic-undemocratic	Activist-less outspoken (13.10%)	Democratic-undemocratic	Activist-less outspoken	Politically engaged-not politically engaged	Activist-less outspoken (11.39%)
(12.24%)		(11.14%)	(14.13%)	(14.38%)	
Activist-less outspoken (9.96%)	Democratic-undemocratic (12.99%)	Having integrity-not having integrity	Like me-unlike me (12.93%)	Activist-less outspoken (12.46%)	Democratic-undemocratic (10.27%)
		(10.10%)			
Having integrity-not having integrity	Informed-ill informed (9.82%)	Informed-ill informed (9.84%)	Politically engaged-not politically engaged	Like me-unlike me (10.72%)	Like me-unlike me (9.97%)
(9.81%)			(10.80%)		
Informed-ill informed	Like me-unlike me	Like me-unlike me	Informed-ill informed	Informed-ill informed	
(9.71%)	(9.44%)	(9.76%)	(9.31%)	(10.46%)	
Like me-unlike me (9.66%)	Having integrity-not having integrity			Stressed-unstressed (9.48%)	
	(9.24%)				
	Politically engaged-not politically engaged				
	(9.06%)				

**Democratic—Undemocratic**. The construct “democratic-undemocratic” appears particularly important for the Leave group. This was reflected in discussions with participants, who strongly expressed their feelings that the referendum outcome was not being honored and that this was undemocratic.

**Having Integrity—Not Having Integrity**. “Having integrity—not having integrity” appears to be important in all Leave groups but only in the Remain fused group. This may reflect the strongly expressed opinions of Leave activists regarding, for example, Members of Parliament (MPs) who voted in parliament differently to the referendum vote of their constituency. These MPs were referred to as “traitors” and would be discussed in tandem with discussions of the democracy of the Brexit process.

**Activist—Less Outspoken**. In contrast, for the Remain group the “activist-less outspoken” construct was critical, perhaps reflecting the importance of demonstrating.

**Like Me—Unlike Me**. “Like me-unlike me” also discriminated highly between elements in the Remain group, suggesting that it is particularly important to Remain activists whether a person is like them or not. This perhaps reflects an increased polarity in thinking about fellow and opposition activists, as well as the importance of homogeneity to the Remain group.

**Informed—Ill Informed**. “Informed—ill-informed” appears to be important to all groups other than the Remain fused group. This was surprising, as the Remain activists were a very informed group (in discussion with the author they would demonstrate a sound knowledge of the detailed issues involved in the Brexit debate).

#### Correlation of Measures

Willingness to undertake extreme behavior was positively correlated with the distance of opposition activists from the ideal self, τ(60) = 0.18, *p* < 0.05 (two-tailed). This suggests, perhaps unsurprisingly, that individuals who were more willing to undertake extreme behavior were those who construed opposition campaigners most negatively.

Time since becoming an activist was positively correlated with the (hypothetical) likelihood of:

- sacrificing one's life to save one fellow activist whilst abandoning five opposition activists to their death, τ(63) = 0.23, *p* < 0.05 (two-tailed).
- undertaking political violence if the group were dismantled, τ(63) = 0.20, *p* < 0.05 (two-tailed).

This suggests that time spent with fellow demonstrators increases the willingness to undertake hypothetical extreme acts of pro-group behavior.

### Interview Data Analyses

Of the 65 participants in the study, 30 took part in the interview, of which there were 14 Leave and 16 Remain participants.

#### Deductive Analysis

Deductive analysis demonstrated support of the constructivist model, as illustrated by the following quotes from participants, presented in relation to the stages of the model that they were considered to represent:

1. The radicalized individual has a history of invalidation of his/her construing, particularly in regard to core aspects of self-construing.

“*I believe in being a patriot, I believe in democracy, and we had a democratic vote and Leave won and Remain lost, so I'm here defending and supporting because day by day everything looks like it's slipping away slowly.”* Leave activist 1005

“*It's a very worrying stress and it's, it's deeply hurtful because I feel like all the best parts of my country are being ripped away by lies”* Remain activist 1019

2. Invalidation can sometimes involve one or more episodes that lead to massive invalidation, and act as “transformative triggers.” This occurs when several superordinate structures, including core constructs, become invalidated in a short period of time. The resultant extreme uncertainty is experienced as intense anxiety, threat and associated emotional responses.

“*I was absolutely angry as I could ever be that they were trying to reverse the democratic vote”* Leave activist 1023

3. The individual with a very undifferentiated (and thus inflexible) construct system may be particularly vulnerable to such invalidation and consequent structural collapse. Individuals with undifferentiated (inflexible) construct systems have a limited view of events. Their construct system cannot easily provide an alternative understanding and they may be particularly vulnerable to construct invalidation.

“*No. Nothing changes me. There is nothing to learn about it.”* Leave activist 1009

“*I was bullied as a kid at school and basically what the Tory party is doing now is bullying the country”* Remain activist 1016

4. His/her radical beliefs, usually drawing upon available social constructions, allow the development of a “turning point” in his or her sense of identity with a more structured and certain view of the world. Following attempts to reconstrue in order to understand their experience, an individual may turn to an ideological framework to restore certainty, reduce anxiety and have a new core role as a member of the group.

“*I've changed a lot with it. I used to be withdrawn but I feel outgoing now and I'm happy with that”* Leave activist 1009

“*I feel more involved now, more happy”* Leave activist 1012

“*Campaigning has made me feel stronger!”* Leave activist 1057

“*Brexit is a religion and ideology”* Remain activist 1017

“*Even if it doesn't affect anything, it helps me, kind of selfishly, to feel more positive about the situation”* Remain activist 1026

“*I felt very strongly about it and rather than worry at home I started to join in and I felt much better for it*” Remain activist 1008

“*I wasn't a European federalist before but I would probably support it now”* Remain activist 1019

5. The development of an extreme negative construction of another group, which may be perceived as responsible for the individual's invalidations, allows further definition of the self by contrast with this group.

“*I think they're traitors, I've always thought that. I'll tell them to their face they are traitors … I can't stand none of them … They are snakes, they are slimy”* Leave activist 1005

“*They are just rather nasty people aren't they … they want to destroy a nation”* Leave activist 1025

“*They've got no arguments, they are fools”* Remain activist 1019

“*they are actually quite unpleasant … some of them are really quite nasty pieces of work”* Remain activist 1027

6. The individual's radical constructions are validated by contact with others who share similar views, often coupled with constriction of their previous social world to avoid further invalidation. Radicalized individuals will often reduce their social contacts to those who are their primary source of validation.

“*it's not just similar values, it's principles … they are my favorite sort of people”* Leave activist 1025

“*the other people who were here were very similar to me in terms of you know, the kind of people they were”* Leave activist 1010

“*If there was no protest movement … I would probably not be standing outside on my own”* Leave activist 1022

“*I see us Leavers as family, it's a great thing*” Leave activist 1041

“*We're there for each other, like a family. We're a tribe!”* Leave activist 1055

“*You do pal up with people here … because they have similar views you actually quite like”* Remain activist 1018

“*it took a little while after the referendum result to realize that there was actually a campaign going on that I could be part of it. I think I joined the [town] for Europe group and then came up here”* Remain activist 1015

“*this is my family, this lot … I feel really close* [eyes welling up with tears]. *I get very emotional when I think about this lot”* Remain activist 1019

“*we are family”* Remain activist 1003

7. The likelihood of acting upon radical beliefs, including violent actions, is greater in those individuals in whom beliefs in such actions provide the greatest increment in the structure of his/her view of the self. Taking extreme actions may enhance the structure and certainty provided by their new role.

“*I'm getting up in the morning … I'm coming up there … that's what I mean I'm addicted”* Leave activist 1021

“*it's changed me from … a passive or indifferent Eurosceptic to being a confirmed active Brexit protester”* Leave activist 1022

“*I am quite happy to put in 6, 8, 10 h you know, cause what else would I do?*” Remain activist 1004

“*People aren't activists when they, join but they get more involved as they get more confident. It happens in steps. It starts with posting leaflets, then coming to meetings, getting more involved, then marching and demonstrating”* Remain activist 1033

8. Reconstruing of violence as acceptable may be necessary if the person is to engage in such acts without guilt (and indeed to experience guilt for not engaging in them).

“*I think those MPs who voted different to their constituents are going to get hurt. I can see people hurting them. I can see* [female MP] *getting hurt, being attacked … I actually think they deserve it. Traitors! … If anyone gets hurt I don't care. I'll push them over. I threw water over* [high profile Remain campaigner] *the other day”* Leave activist 1005

“*If anyone hurts someone in our group …we all jump in”* Leave activist 1021

“*well you know we take flags to places and um somebody came along and tried to help themselves to one … so a little scuffle ensued”* Remain activist 1004

“*dangerous times, but I still believe you should stand up for what you believe in”* Remain activist 1007

9. His/her radical view of the world may be shored up by “hostility,” in Kelly's (1955) sense of extorting evidence for the individual's constructions. Kelly (1955) describes hostility as when an individual is unable to revise their construct system to understand new events and instead forces the evidence to fit.

“*The Germans are all-powerful in the EU, they are simply developing the Fourth Reich”* Leave activist 1009

“*I thought no … you've got to respect nearly half of the population was a clear vote to remain”* Remain activist 1016

10. Similar processes may operate in members of the “other” group, creating a vicious cycle of extreme construing based on mutual validation of extreme negative views of the other.

Leave and Remain demonstrated similar processes, as evidenced here in the narrative and in the quantitative measures.

#### Inductive Analysis

Thematic analysis identified different themes for the Leave and Remain activist groups.

**Leave Activists**. Five principal themes were observed. These were dissatisfaction with the EU, destruction of democracy, the threat of immigration, the disregarding of their views and the Second World War.

***Dissatisfaction With the European Union***. Leave were dissatisfied with the EU and did not consider it beneficial for the country. This focused upon EU influence in limiting British government decision-making and the lack of accountability of EU officials.

“*because I'm fed up with the way we get told to do things and the way that the EU run things”* Leave activist 1012

“*very good for Britain to come out… can run our own office... they can use the money to invest on our local issues like education.. can invest in the police”* Leave activist 1024

“*thinks it can dictate to all of the other countries on the continent this, just too radical”* Leave activist 1025

“*and then the commission isn't elected and doesn't actually propose the legislation and then you've got all the backhanders”* Leave activist 1025

***Destruction of Democracy***. Leave activists were frustrated by the referendum vote not being delivered and considered this an erosion of democracy.

“*we had democratic votes and Leave won and Remain lost, so I'm here defending”* Leave activist 1005

“*because that's what I voted for, that's what I expect you know ‘cause I'm, I do believe in democracy right and because you're not gonna deliver the vote well that's not democratic”* Leave activist 1021

“*What did Emmeline Pankhurst fight for?”* Leave activist 1011

“*A big part of it is the fact that you have had a vote, had an outcome and it's not being followed”* Leave activist 1023

“*I don't understand why they would want to reverse a democratic vote which is what they are trying to do”* Leave activist 1023

***The Threat of Immigration***. Leave participants believed that membership of the EU resulted in unrestricted numbers of immigrants, which they considered a threat to the United Kingdom.

“*um I've been on all these sites where I've seen this happen and we are we are being totally invaded by the open-door policy”* Leave activist 1005

“*and it's full of people coming over, doing as much overtime as they can and then going back and getting all everything … no for nothing plus their income tax back and I've claimed my parents had no money, but they claim for nothing... they've worked hard”* Leave activist 1011

“*they will say those employers will say things like we've had British people here they don't work out or whatever, that is at best a distortion… a British person will stand up for themselves and might stand for something that … whereas certainly people er from our other countries not knowing their rights, they will not stand up for themselves”* Leave activist 1022

***The Disregarding of Their Views***. Despite winning the referendum, Leave activists outside the Houses of Parliament considered themselves unheard and disrespected by the government, Remain campaigners and the police.

Government.

“*we got politicians we can't trust”* Leave activist 1005

“*well yeah it's* [parliament] *basically a bunch of snobs who look down on us peasants as people who are too stupid to know what they're voting for”* Leave activist 1025

“*It's all some sort of game they* [parliamentarians] *can, they can walk out. That's why they have so much fun. We can't, everything is on the line for us”* Leave activist 1025

Remainers.

“*we've been involved in big heated arguments with the Remainers … we have been told to go home, we have been told to stop being stupid”* Leave activist 1012

Police.

“*and he did it again with somebody else and they called the police, but the police did nothing because I think they were just trying to keep things calm, because they were trying to push us away from that area down there away from where all the television”* Leave activist 1010

“*So we've started to come up and the amount of abuse we're getting it's unreal and the actual police are not doing a single thing about it which we get all the time”* Leave activist 1021

***The Second World War***. Leave activists believed that the sacrifices of the Second World War were, in part, to preserve the sovereignty of the British nation.

“*My father was in the Second World War, he was a fireman and saw awful things because we come from Lincolnshire, where it's ‘Bomber County.' What did they fight for?”* Leave activist 1011

***Disassociation From far-right Protestors***. Far right protestors, such as the ‘yellow vests” were seen as too extreme. There was concern amongst Leave activists that they and their cause would be associated with this extreme group and their violence.

“*Yellow vests are a bit more militant*” Leave activist 1022

**Remain Activists**. Five principal themes were found. These were the benefits of the European Union, awareness of Britain's place in the world, dissatisfaction with political processes, personal experiences and the Second World War.

***Benefits of the European Union***. This includes ensuring peace, providing equality through opportunity, and ensuring standards.

“*the EU you can't argue with the fact that it's the biggest peace project for the last 70 years”* Remain activist 1016

“*I remember the Berlin wall coming down … and I think it was just such a relief to have that barrier gone... we were all joined together… peace and cooperation”* Remain activist 1008

“*I'm from a working-class background … I feel that by exiting the European Union er opportunities for people like me are going to start shrinking”* Remain activist 1020

“*I think Europe has an ethos that says, that says something about fairness and opportunity for all”* Remain campaigner 1003

“*The European Union has actively, you know … doing things like roaming charges.. putting in employment rights protection, workers' rights and all of these little things, they all help the little person on their feet”* Remain activist 1016

“*The EU has done so much for environmental standards and food standards”* Remain activist 1007

***Awareness of Britain's Place in the World***. Remain activists believed that Britain could not stand alone on the world stage and would benefit from cooperation with others.

“*A lot of people over here seem to have this island mentality … Europe bad, old British is good and harking back to the Empire and all this nonsense, when we should be looking ahead and looking forward and trying to be positive about our neighbors and you know, we're all here to work together, we're all on the same planet”* Remain activist 1016

“*it's just realizing with such a small little island … that the world doesn't operate, you cannot operate on your own anymore. You have to be melded in into some sort of group identity or system”* Remain activist 1007

***Dissatisfaction With Political Processes***. Remain were dissatisfied with the government and the referendum process.

“*Change the government to a government that know what it's doing”* Remain activist 1004

“*If we have another year, the members of parliament are still going to be sat there.. in a year's time.. Wrapped up in circles in the same position.. Not finding a solution”* Remain activist 1007

“*there was a court case challenging the validity of the referendum … and the judges found that that the electoral law had been broken and um if it hadn't been, if the government were compelled to follow the results of the referendum, rather than it having been advisory.. it would have been null and void”* Remain activist 1019

“*a second referendum which isn't marred in cheating and um lies like the last one and we get a fair pop at things”* Remain activist 1016

***Personal Experiences***. Remain frequently referred to their personal experiences and motivations.

“*my real concern is that my children won't have the same opportunities”* Remain activist 1017

“*I am an EU lawyer… I advise UK business in particular on EU state aid law*” Remain activist 1019

“*I live in the West Country particularly into Cornwall where there are deprived areas”* Remain activist 1019

***The Second World War***. Remain activists believed membership of the EU would ensure peace, in contrast to the experiences of the Second World War.

“*my father was a Jewish refugee and his parents died in concentration camps and I see a lot of what Brexit is about is the rise of the Far Right, so that frightens me”* Remain activist 1017

“*my grandparents were worried about Hitler but believed the German people would stop him so they didn't do anything. But the German people didn't do anything and my grandparents, they died in a concentration camp. So, I can't do nothing now. The Far Right is active again and I can't let that happen again”* Remain activist 1033

“*what my father did after the war, he was sent to Berlin to help reconstruct Europe”* Remain activist 1004

***Fear of the Far Right***. Remain activists were concerned with the influence and impact of the Far Right on British Society.

“*I do fear the far right …but their issue isn't necessarily with the EU.. they're just against … multiculturalism and that's dangerous”* Remain activist 1007

The narrative data thus reveal the Leave group as primarily concerned with their dissatisfaction with the EU and the destruction of democracy. In contrast, the Remain group's principal concerns were centered on the benefits of the EU and dissatisfaction with the British government. Themes the groups had in common included the Second World War and inequality, although these varied in character. For Leave, inequality referred to that between British citizens and foreign nationals whilst for Remain, it concerned the inequality between social classes.

## Discussion

Brexit activists demonstrating outside the British Houses of Parliament were studied *in situ* to examine their potential for pro-group extreme behavior. Results were similar for both Leave and Remain, with the majority of activists identified as “fused” to their group and, if so, being more likely to undertake hypothetical extreme behavior than non-fused participants. Constructivist measures indicated that becoming an activist provided individuals with a clearer and more positive view of themselves. Opposition activists were construed more negatively and extremely than fellow activists, and this construal was associated with an increased willingness to undertake extreme pro-group behavior.

### The Brexit Context

For three-and-a-half years, the United Kingdom was in a state of flux as the nation and its government struggled to facilitate the Brexit vote of 2016. For many, families, friends and romantic partners would become estranged. For others, Brexit would be a taboo subject, unspoken for fear of its divisive nature and it was abundantly evident that allegiances with Leave or Remain contributed to the identity of a large proportion of the British population (Hobolt et al., 2018; Evans and Schaffner, 2019).

Whilst media representations were inaccurate [age, rather than class or geography, was the greatest vote determinant (IPSOS, 2016)], the nation had indeed been split into dichotomous, polarized groups. According to Turner et al. (1989), polarization occurs when group members conform to an extreme group-norm, as exemplified in the Brexit-based racial violence of 2016 (Burnett, 2017), and subsequent years saw many clashes between Leave and Remain, including those observed by the first author. As Hughes (2019, p. 88) points out in his book *Brexit Psychology*, “As things spiral further, soon it feels right to start defending your group from rivals. And of course, often the best form of defense is attack.” Following Brexit, strong emotions have continued to be evident in, for example, social media debates involving participants not just from the UK but also various other countries, and including expressions of Schadenfreude by those who perceived Brexit as unjust at subsequent misfortunes suffered by the UK (Cecconi et al., 2020).

### Leave and Remain Activist Groups

Whilst differences were observed in the characteristics of Leave and Remain, the more striking finding was their similarity. This included the proportion described as fused, their construing processes and their willingness to undertake hypothetical extreme behavior. Importantly, such similarities indicate that the findings were a function of group membership rather than political stance and are therefore relevant to activism beyond the Brexit campaign. These similarities also point to optimism for resolution of the UK's schism. Commonality between groups reduces their distinctiveness and can help develop more positive out-group attitudes and behaviors (e.g., Dovidio et al., 1997; Schori-Eyal et al., 2019). By recognizing the similarity in their core constructs, disparate groups can recategorize into one overarching collective with a new superordinate identity in which the in-group now encompasses former out-group members (Dovidio et al., 2000). Given the high level of commonality in this study, reconciliation should be possible between those who supported Leave or Remain. In fact, with the onset of Covid-19, a superordinate identity of “plagued nation” was observed within months of the Brexit departure. As the country entered lockdown, the entire nation initially became united in support of one another (e.g., Daily Mail, 2020) and discussion of Brexit halted. However, as Denning and Hodges (2021) suggest, high identification with a group and corresponding “counter-projection” (seeing the opposite of oneself in others) makes it more difficult to find common ground. It is therefore important to be mindful of identity fusion in attempts to reduce political conflict.

Whilst commonalities enable constructive dialogue, each group has a distinct identity and those of Leave and Remain have been well-researched (e.g., Hobolt et al., 2018; Manners, 2018; Peitz et al., 2018; Swami et al., 2018; Virdee and McGeever, 2018). Distinctive characteristics were also found in this study. Perhaps predictably, dissatisfaction with the EU was an important theme emerging from the Leave narrative, as were the destruction of democracy and the threat of immigration. In contrast, Remain found the benefits of the European Union, awareness of Britain's place in the world and personal experience held particular significance. These themes informed and shaped the groups' political stances. Interesting parallels also existed between the groups. Whilst similar, these were characterized in different ways. For Leave, inequality referred to that between British citizens and foreign nationals but for Remain, it concerned the inequality between social classes. Similarly, both groups emphasized the importance of the Second World War to the debate. Leave felt that the sacrifices involved should be respected and enshrined in autonomous British sovereignty, whereas Remain believed that the European Union was essential to ensure the preservation of peace. Whilst Remain described positive experiences in other countries and cultures, Leave spoke exclusively about their lives in the United Kingdom. Such similarities and variations in themes are perhaps characteristic of polarized groups on opposing sides of a single issue.

These differences were echoed in the constructs identified as most salient from repertory grid analyses. For Leave demonstrators, the most important construct was “Democratic—undemocratic.” They had assumed that the referendum outcome would result in an expeditious departure from the EU. However, Parliament's repeated rejection of withdrawal proposals destroyed their belief in democracy (and led to vehement shouting of “Traitor” at passing MPs). In comparison, for Remain, the construct “Activist—less outspoken” held most significance. This reflected their belief that protest was the only way to stop Brexit.

The Leave vote may also have been associated with collective narcissism (Marchlewska et al., 2018). Inflated belief in in-group greatness is contingent on external recognition of the in-group's worth and is associated with the success of populist movements (Golec de Zavala and Keenan, 2020). It involves an exaggerated perception of threat and a propensity for hostile responses (Cichocka and Cislak, 2020). As observed in the Leave narrative, the threat of immigration was a substantial concern and was encased in hostile terminology.

“*we are being totally invaded by the open-door policy*” Leave activist 1005

### Identity Fusion

Over seventy percent of the study population was found to be fused to their group. This is considerably greater than that observed in Swann et al.'s (2009) original study (41%), and likely reflects the study being undertaken *in situ*, where committed group members were actively demonstrating. In addition, both campaign groups were present throughout, either as demonstrators or as passers-by engaging in debate. The presence of an opposing group challenges the other, heightening in-group allegiance.

Hypothetical extreme pro-group behavior was more prevalent amongst the study's fused individuals, who tended to score more highly on all measures. This included an elevated willingness to fight for the group and to sacrifice their life to save a fellow group member. The latter was heightened when there was an option to sacrifice themselves for an opposition activist, again highlighting the effect of the presence of the opposing group and demonstrating the centrality of group interaction. This is also illustrated by participants being increasingly more willing to sacrifice their life and undertake political violence with increased time spent as a campaign group member. Time with fellow activists likely reinforces both inter- and intra-group dynamics and, as a result, the group becomes increasingly important to the individual, to the point where they are willing to undertake hypothetical extreme acts. The significance of the group is markedly emphasized in fused participants being more likely to anticipate undertaking political violence if the group were to be dismantled. That they were less likely to do so to achieve the group's aims is noteworthy, further clarifying that it is the group itself, rather than its political ambitions, that has the greater influence on extreme behavior. A recent study by Reiter et al. (2021) involving analysis of narrative data surrounding radicalization and deradicalization has further supported the importance of social identity and social belonging in both of these processes.

The willingness to undertake extreme pro-group acts may appear surprising considering the personal cost. However, it is the individual's extraordinary relationship with their group, rather than its aim, that motivates these actions. As Swann and Talaifar (2018) suggest, some fused individuals believe that even if they should die, they would continue to live on in the group.

As can be seen, the study's findings support the concept of identity fusion, its presence in activist populations and its association with extreme behavior. In addition, the verbal measure of identity fusion (Gómez et al., 2011) was supported and found to be predictive of scores on Swann et al.'s (2010b) measure of willingness to fight. This is valuable as it indicates a potential for the verbal fusion measure, with lower face validity, to assess the likelihood of pro-group behavior. It could therefore be usefully employed in programmes aimed at the prevention of extreme actions.

### Processes of Construing

Becoming an activist provided individuals with clearer and more positive views of themselves. This was shown by an increased closeness to the individual's ideal self and decreased conflict in self-construing (from repertory grid analyses). This more positive and coherent self-view likely motivates individuals to begin and maintain activism.

Reflecting the polarization of the Brexit debate, opposition activists were construed in a substantially more negative and conflicted way than fellow campaigners. Notably, individuals found opposition activists to be more salient than fellow campaigners. This may indicate that, as described in social identity theory (Tajfel, 1978; Tajfel and Turner, 1979), extreme negative construing of the opposing group allows individuals to construe themselves more positively in comparison. This may be another motivation for activism and would be particularly relevant to Brexit demonstrators, who were in close proximity to the opposing group throughout. That these findings were amplified in fused participants highlights, once again, the importance of the opposing group in group effects.

Perhaps consistent with the view that “affective polarization” of one's own and an opposition group may have toxic consequences in, for example, leading to erosion of democratic norms and dehumanization of the other group (Moore-Berg et al., 2020; Arbatli and Rosenberg, 2021; Kingzette et al., 2021), those individuals who viewed opposing activists more negatively were found to be more willing to undertake hypothetical extreme pro-group behavior. Whilst this may be unsurprising, it suggests that repertory grid technique could be an effective measure to develop as part of an assessment tool, for example in preventative programmes. Grids have low face validity and are thus able both to access construing at a low level of awareness and potentially to provide an indicator of likelihood of extreme pro-group behavior.

### Constructivist Model of Radicalization

Repertory grid and interview data support the constructivist model of radicalization (Winter and Feixas, 2019). For example, the degree of tightness of the individual's construct system was consistent throughout the population and was also evident in activists' narratives. It may indicate a certain inflexibility associated with activism. As Winter and Feixas (2019, p. 4) suggest “The individual with a very undifferentiated (and thus inflexible) construct system may be particularly vulnerable [to radicalization].”

The interview provided vivid examples of activists' radical beliefs reducing anxiety by providing a more certain world view. This both reinforces and explains the repertory grid data which demonstrated that becoming an activist had a positive impact.

The study also supported the model's proposition that “extreme negative construction of another group …allows further definition of the self” (Winter and Feixas, 2019, p. 4). Brexit activists' construing of the opposing group was extremely negative (in both repertory grid and interview data), which would have enhanced the positivity and clarity of their self-view and identity. That a sense of self and self-esteem are achieved, at least in part, by negative viewing of an out-group may explain why opposition activists were construed as so much more meaningful than fellow campaigners. Tajfel and Turner's (1979) discussion of social hierarchies may also be relevant here. The lower a group's status, the less its contribution to a positive social identity. Group members react to this in several ways including redefining the comparison group in a more negative manner. Remain activists appeared to consider themselves of superior morality: “*Unlike Leave, we want peace across Europe”* [Remain activist 1026]. A threat to this superior, positive comparison would require defending. In contrast, Leave activists, despite having won the referendum, considered themselves the underdogs. They were consistently lower in demonstrator numbers and the “Westminster political bubble” was clearly pro-Remain. As a result, Leave supporters appeared to redefine the inferior-superior comparison by attacking Remain supporters on the morally unambiguous issue of democracy: “*They think they're right but they're making a mockery of democracy”* [Leave activist 1055]. These standpoints could also be seen in terms of Bandura's (1991) moral disengagement theory. The theory describes several mechanisms including, as here, moral justification. Similar findings were also observed in a study of polarized aggressive responses to an online sexist meme by Paciello et al. (2021).

That “Radical constructions are validated by contact with others who share similar views” (Winter and Feixas, 2019, p. 5) is also supported by the study's findings. Polarized construing is enhanced by informational influence (Turner et al., 1989). This includes the concept of the echo chamber, in which discussions are reduced to involve only those who are of a similar opinion. As a result, only identical and complementary arguments are heard and the viewpoint is reinforced and polarized, thereby validating the individual's (radical) constructions. This was evident in the data and in discussions between demonstrators at the Houses of Parliament. That individuals who had spent longer as activists were more willing to undertake pro-group behavior suggests that the more time spent with fellow activists, the greater the opportunity to reinforce and validate their political views.

Narrative data also provided an indication of reconstruing violence as acceptable. Several individuals from both Leave and Remain appeared to justify violent actions. Bandura (1991) also suggests that individuals morally justify harmful behavior by reconsidering it as essential to the attainment of a noble goal, as similarly described in the constructivist model and observed in this study.

The constructivist model suggests that the “radical view may be shored up by hostility” (Winter and Feixas, 2019, p. 5). The PCP concept of hostility is to extort evidence for a “social prediction which has previously been recognized as a failure” (Kelly, 1955, p. 375). For example, a Leave activist may construe a particular Remainer as a “bad” person but has no evidence to support this. The Leave activist may therefore behave in a manner to elicit such evidence and validate the negative construction. They could be verbally or physically aggressive toward the Remainer, provoking an equally aggressive response, and thereby fulfilling their prediction of the Remainer as “bad.” This type of hostility was often observed during the study and in the interview. It has also been well-documented in the press (e.g., Osborne, 2019).

That “Similar processes may operate in members of the “other group,” creating a vicious cycle of extreme construing” (Winter and Feixas, 2019, p. 5) is well-supported by the study in the abundance of similarity in the processes of Leave and Remain groups. This ranged from the willingness to undertake hypothetical extreme acts to the close correlation of repertory grids and similarities in interview narratives, to the levels of identity fusion.

The constructivist model of radicalization (Winter and Feixas, 2019) is supported by the study and Brexit activists' construing was consistent with that observed in studies of radicalization in Salafist Muslims (Winter and Muhanna-Matar, 2020) and analyses of the narrative of Anders Breivik (Winter and Tschudi, 2015), which supported the development of the model.

As one study participant put it: “*I consider myself a radical. My job is to radicalize others”* (Participant 1003).

### Radicalized Terrorism

Researchers such as Horgan (2017) have highlighted the lack of a terrorist profile in terms of educational level, personality traits or psychopathology although some individual and situational factors may contribute (e.g., Moghaddam, 2005). Rather, it tends to be the ordinary citizen who becomes a terrorist. Whilst Brexit activists are far from terrorists, the process in which some of these “ordinary citizens” become willing to undertake violence may follow the same pathway as individuals who progress much further. In this way, activist and campaign groups may provide a useful insight into the subject of radicalization and its often tragic consequences, a psychological and socio-ecological approach to which is likely to be more productive than one that is purely security-driven (Miconi et al., 2021).

## Strengths and Limitations

Whilst it presented many challenges, including aggressive demonstrators and the British weather, the real-world population of the study provided a great strength. Here was a population in which some individuals had been involved in violent actions (e.g., Busby, 2019). Thus, people who had undertaken actual extreme pro-group acts contributed to study data.

The novel integration of several methodologies was of equal value. It enabled the obtaining of data at both the group and individual level, thus providing a greater depth of understanding. Furthermore, whilst social identity research could be criticized for its use of self-reporting surveys, PCP techniques, particularly the repertory grid, are less vulnerable to dissimulation and more likely to access aspects of construing at low levels of awareness. Whilst PCP provides a comprehensive, detailed and far-reaching approach, social identity theory acknowledges the importance of group membership and, particularly, the role groups play in our sense of self and others.

The sample size was smaller than desired, particularly for subgroup analyses. However, statistically significant results were still obtained at this level.

The sampling method was opportunistic and therefore activists were not selected entirely at random. Activists who knew previous participants were more likely to take part as they observed the trust established between the researcher and a fellow group member.

## Conclusion

The Brexit demonstrations of 2019–2020 provided a valuable opportunity to investigate identity fusion theory, the constructivist model of radicalization and the prediction of extreme pro-group behaviors.

A large proportion of the campaign groups were identified as fused and demonstrated an increased willingness to undertake personally costly, pro-group acts, including self-sacrifice. The constructivist model (Winter and Feixas, 2019) was supported and highlighted the progression of some activists along the pathway of radicalization. In line with previous research (Winter and Muhanna-Matar, 2020), Brexit activism provided individuals with a more positive and certain sense of self.

Given the considerable number of fused individuals found in the study, it would be worthwhile exploring identity fusion in other activist populations. Moreover, there is potential to develop the study measures for use within programmes involved in the prevention of radicalization-based violence. Both the repertory grid (Winter, 2011) and the measure of identity fusion (Gómez et al., 2011) have lower face validity, highlighting their usefulness as effective tools in assessment and prevention.

The innovative combination of theoretical backgrounds provided a valuable insight into the thinking and potential actions of activists. Given the severity of the violent impact of extremist activists around the globe, the findings of this study make an important contribution to the field.

## Data Availability Statement

The raw data supporting the conclusions of this article will be made available by the authors, without undue reservation.

## Ethics Statement

The studies involving human participants were reviewed and approved by the University of Hertfordshire Health, Science, and Engineering and Technology Ethics Committee with Delegated Authority. The patients/participants provided their written informed consent to participate in this study.

## Author Contributions

CM, DW, and SS were responsible for the design of the study and the structure of the paper. CM was responsible for data collection and for writing an initial draft of the manuscript, into which feedback from DW and SS was incorporated. BB was primarily responsible for the inductive analysis. All authors contributed to the article and approved the submitted version.

## Funding

Funding for this publication was provided by the University of Hertfordshire.

## Conflict of Interest

The authors declare that the research was conducted in the absence of any commercial or financial relationships that could be construed as a potential conflict of interest.

## Publisher's Note

All claims expressed in this article are solely those of the authors and do not necessarily represent those of their affiliated organizations, or those of the publisher, the editors and the reviewers. Any product that may be evaluated in this article, or claim that may be made by its manufacturer, is not guaranteed or endorsed by the publisher.

## Footnotes

^1^Article 50 is the legal mechanism for a member state to leave the European Union and withdraw from its treaty obligations.
